# Utilizing Real-time Technology to Assess the Impact of Home Environmental Exposures on Asthma Symptoms: Protocol for an Observational Pilot Study

**DOI:** 10.2196/39887

**Published:** 2022-08-02

**Authors:** Sharmilee Nyenhuis, Emily Cramer, Matthew Grande, Luz Huntington-Moskos, Kathryn Krueger, Olivia Bimbi, Barbara Polivka, Kamal Eldeirawi

**Affiliations:** 1 Section of Allergy and Immunology Department of Pediatrics University of Chicago Chicago, IL United States; 2 Biostatistics & Epidemiology Core Health Services & Outcomes Research Children’s Mercy Research Institute Kansas City, MO United States; 3 School of Medicine University of Missouri-Kansas City Kansas City, IL United States; 4 College of Medicine University of Illinois Chicago Chicago, IL United States; 5 School of Nursing University of Louisville Louisville, KY United States; 6 School of Nursing University of Kansas Kansas City, KS United States; 7 College of Nursing University of Illinois Chicago Chicago, IL United States

**Keywords:** asthma, home environment, ecologic momentary assessment, air quality, spirometry

## Abstract

**Background:**

It is estimated that over 60% of adults with asthma have uncontrolled symptoms, representing a substantial health and economic impact. The effects of the home environment and exposure to volatile organic compounds (VOCs) and fine particulate matter (PM_2.5_) on adults with asthma remain unknown. In addition, methods currently used to assess the home environment do not capture real-time data on potentially modifiable environmental exposures or their effect on asthma symptoms.

**Objective:**

The aims of this study are to (1) determine the feasibility and usability of ecological momentary assessment (EMA) to assess self-report residential environmental exposures and asthma symptoms, home monitoring of objective environmental exposures (total VOCs and PM_2.5_), and lung function in terms of forced expiratory volume in 1 second (FEV_1_%); (2) assess the frequency and level of residential environmental exposures (eg, disinfectants/cleaners, secondhand smoke) via self-reported data and home monitoring objective measures; (3) assess the level of asthma control as indicated by self-reported asthma symptoms and lung function; and (4) explore associations of self-reported and objective measures of residential environmental exposures with self-reported and objective measures of asthma control.

**Methods:**

We will recruit 50 adults with asthma who have completed our online Global COVID-19 Asthma Study, indicated willingness to be contacted for future studies, reported high use of disinfectant/cleaning products, and have asthma that is not well controlled. Participants will receive an indoor air quality monitor and a home spirometer to measure VOCs, PM_2.5_, and FEV_1_%, respectively. EMA data will be collected using a personal smartphone and EMA software platform. Participants will be sent scheduled and random EMA notifications to assess asthma symptoms, environmental exposures, lung function, and mitigation strategies. After the 14-day data collection period, participants will respond to survey items related to acceptability, appropriateness, and feasibility.

**Results:**

This study was funded in March 2021. We pilot tested our procedures and began recruitment in April 2022. The anticipated completion of the study is 2023.

**Conclusions:**

Findings from this feasibility study will support a powered study to address the impact of home environmental exposures on asthma symptoms and develop tailored, home-based asthma interventions that are responsive to the changing home environment and home routines.

**Trial Registration:**

ClinicalTrials.gov NCT05224076; https://clinicaltrials.gov/ct2/show/NCT05224076

**International Registered Report Identifier (IRRID):**

DERR1-10.2196/39887

## Introduction

### Background

Asthma is a disease of chronic airway inflammation that affects 1 in 12 adults in the United States [1]. Despite effective treatments for asthma, it is estimated that over 60% of adults with asthma have uncontrolled symptoms [2]. Poorly controlled asthma increases the risk of severe asthma exacerbations, which may result in missed workdays, emergency room visits, and hospitalizations [3].

Adults usually spend the majority of their day indoors (~15 hours/day) [4,5], and during the COVID-19 pandemic, the number of hours spent at home increased due to lockdown orders and personal precautions. Environmental triggers of asthma identified in residential settings include aeroallergens (eg, dust mites, animal dander), moisture, tobacco smoke, particulate matter (PM), volatile organic compounds (VOCs), and nitrogen dioxide [3,6,7]. In adults with asthma, indoor residential air quality has been associated with decreased general health, airway inflammation, asthma exacerbation, and decreased lung function [8-12]. In particular, there is strong evidence suggesting that indoor VOCs, especially aromatic and aliphatic compounds, are associated with increased asthma symptoms [12].

VOCs are widely used as ingredients in household products, including cleaning, disinfecting, cosmetic, degreasing, and hobby products. Early in the pandemic, it was recommended to disinfect surfaces with a US Environmental Protection Agency (EPA)–registered household disinfectant [13]. While the effects of disinfectants on adults with asthma have been widely explored in occupational settings, less is known about their impact in the home environment. Our team examined disinfectant use by adults with asthma using an online global survey between May and October 2020. We found a dramatic increase in household disinfectant use during the pandemic, and this correlated with poorer asthma control [14]. What is not known is the extent to which adults with asthma routinely engage in these types of practices, how often they clean/disinfect, and what impact these practices have on their asthma. In addition, data on how these environmental exposures impact asthma symptoms in real time are not available.

PM are small particles or liquid droplets that include acids, organic chemicals, metals, soils, dust, and allergens [15]. Common indoor sources of particulates include vacuum cleaner bags, printers, cooking, secondhand smoke, wood combustion in fireplaces or stoves, dust, pet hair, mold, candles, and outside and biological sources [10,16]. A study found that concentrations of fine particulates less than 2.5 micrometers (PM_2.5_) were highest indoors in homes (versus outdoors or in offices) on weekends when participants wearing personal air quality monitors were at home for longer periods [17], thus supporting the increased potential exposure to particulates during the COVID-19 pandemic. PM_2.5_ have been shown to affect cardiovascular health, anxiety, cognitive function, and respiratory health [16]. However, the effects of indoor PM_2.5_ on asthma are still unclear [12]. Increased frequency and severity of asthma exacerbations, symptoms, hospitalizations, and mortality were associated with increased ambient PM_2.5_ [18-21]. Existing research on VOCs and particulate matter provides a strong scientific premise for further exploration of these exposures in the home. We aim to extend the existing research to develop innovative intervention strategies for those with asthma, thereby addressing the impact of changes in environmental exposures related but not limited to COVID-19 and enhancing our preparedness for future infectious disease outbreaks.

Collecting home air quality data and asthma symptoms typically rely on subjective patient report, which is impacted by recall bias and resource-intensive methods, such as collection of environmental samples from homes. These methods have made it difficult to fully assess the impact of potentially modifiable home environmental exposures on asthma symptoms in real time or provide practical and timely interventions to reduce exposures and disease burden. Ecological momentary assessment (EMA) allows for the collection of time-dependent, real-time data on self-report measures. Thus, EMA reduces potential biases and enhances ecological validity by taking measurements in real time in the settings where they occur, providing high-quality longitudinal data. EMA has been increasingly applied to diseases impacted by lifestyle and outdoor environmental exposures, including studies of asthma-related exposures [3-5]. Yet, there is limited research utilizing EMA to capture in real-time the impact of home environmental exposures on asthma symptoms. To complement EMA-captured subjective data on symptoms, recently developed commercial home environment sensors, such as the Awair system, have been increasingly used to efficiently collect objective and continuous data on multiple measures of home air quality (eg, VOCs, PM_2.5_). These validated sensors and EMA provide real-time environmental exposure data; however, they have yet to be used to examine the home air quality of adults with asthma and its associations with symptoms in real time [22-25].

Accordingly, this study aims to assess the feasibility and usability of EMA to capture the context of real-time behaviors and environmental exposures that impact indoor environments. In addition, this study assesses the feasibility and usability of providing participants with an indoor air quality monitor (Awair Omni) to continuously capture total VOCs and PM_2.5_. This design will allow us to alert participants of high levels of VOCs and PM_2.5_ and collect real-time data on exposure and asthma symptoms. Daily and exposure-related lung function will be measured with a low-cost home spirometer. Finally, we will examine the effect of residential environmental exposures that may be related to time spent at home and the associations between these exposures and asthma control. In this paper, we will describe the study protocol and outline how multiple commercially available technologies that measure air quality, lung function, and EMA were harmoniously blended and refined prior to pilot testing.

### Conceptual Framework

**Figure 1:**
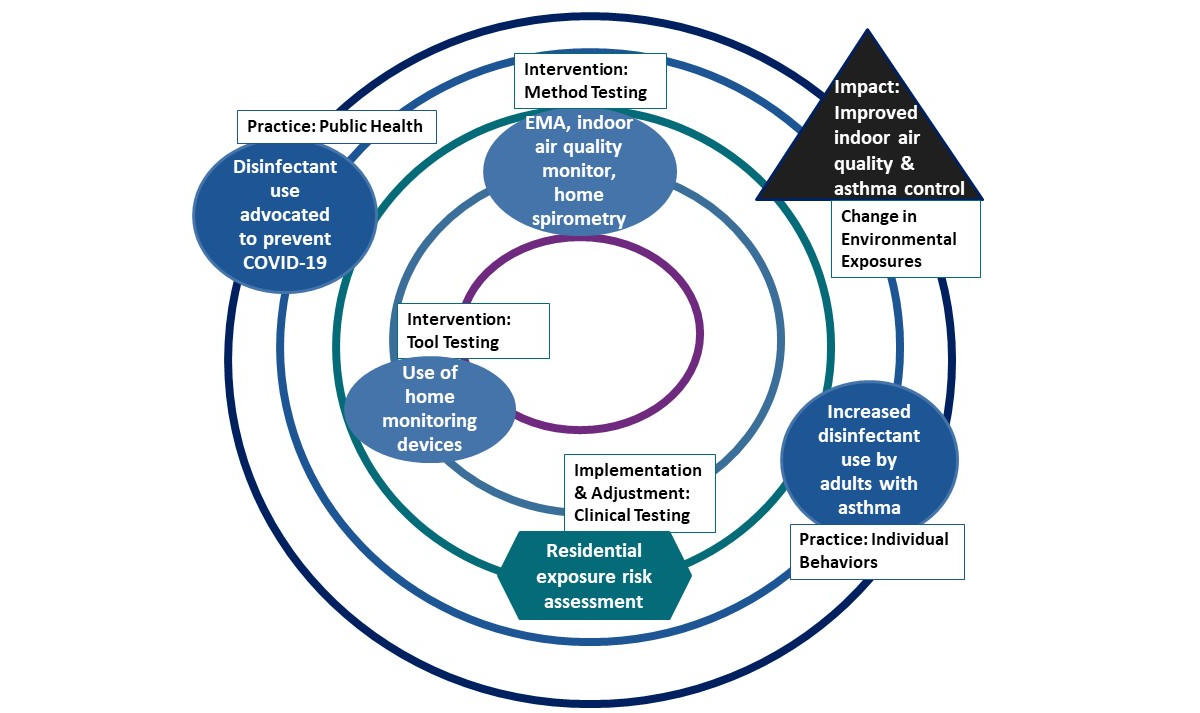
Application of the National Institute of Environmental Health Sciences (NIEHS) Translational Research Framework. EMA: ecological momentary assessment.

Research efforts that focus on indoor home environments are essential to elucidate the possible extent of exposures that can impact asthma outcomes and further exacerbate the risk of hospitalization in a time when home and health care resources continue to be strained. We illustrate the goals of our feasibility study and the impacts we intend to accomplish using the Translational Research Framework [26,27]. This feasibility study will assess residential exposures of disinfectants and particulates with the goal of reducing exposures and improving asthma outcomes (Figure 1).

**Objectives**

The aims of this study are to (1) determine the feasibility and usability of EMA to assess self-report residential environmental exposures and asthma symptoms, home monitoring of objective environmental exposures (total VOCs and PM2.5), and lung function in terms of forced expiratory volume in 1 second (FEV_1_%); (2) assess the frequency and level of residential environmental exposures (eg, disinfectants/cleaners, secondhand smoke) via self-reported data and home monitoring objective measures; (3) assess the level of asthma control as indicated by self-reported asthma symptoms and lung function; and (4) explore associations of self-reported and objective measures of residential environmental exposures with self-reported and objective measures of asthma control.

## Methods

### Project Overview

We will recruit 50 adults with asthma who completed our online Global COVID-19 Asthma Study (GCAS), indicated willingness to be contacted for future research, reported high use of disinfectant/cleaning products, and have suboptimally controlled asthma [14]. Participants will receive an indoor air quality monitor and a home spirometer to measure VOCs, PM_2.5_, and forced expiratory volume in 1 second (FEV_1_%), respectively. EMA survey data will be collected using a personal smartphone and the PiLR Health software platform [28]. Participants will be sent scheduled and random EMA notifications to assess asthma symptoms, environmental exposures, lung function, and mitigation strategies. After the 14-day data collection period, participants will respond to survey items related to acceptability, appropriateness, and feasibility. In addition, a random sample of 20 participants will be interviewed to provide further insights.

### Ethics Approval

All studies were conducted in accordance with the Declaration of Helsinki and the International Conference on Harmonization Good Clinical Practice guidelines and approved by the relevant institutional review boards at the University of Kansas (STUDY00145830), University of Louisville (21.0466), University of Illinois Chicago (2020-0851), and University of Chicago (22-0767).

### Study Components

**Figure 2:**
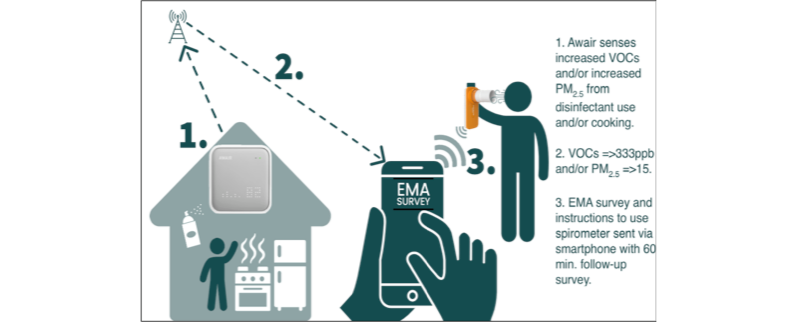
Study components. EMA: ecological momentary assessment; PM_2.5_: fine particulate matter; VOC: volatile organic compound.

Figure 2 shows the study components. The Awair Omni Indoor Air Quality Monitor continuously monitors about 1000 square feet of indoor air for 7 air quality indicators: total VOC, PM_2.5_, temperature, humidity, carbon dioxide, ambient light, and ambient noise [29]. This feasibility study will only focus on VOCs and PM_2.5_ levels (Table 1). The Awair Omni device measures about 4 × 4 × 1.3 inches, is Wi-Fi and Bluetooth–enabled, plugs into a standard alternating current (AC) outlet, and includes an 8-hour battery backup. An air quality reading will be taken every 10 seconds and real-time data uploaded to a dashboard accessed only by research personnel. The Awair Omni proprietary dashboard provides secure communication between the Awair Omni and its dashboard. Data will be exported from the dashboard as a .CSV file [30-34].

**Table 1:** Type and range of air quality measures.

Exposure	Sensor	Detectable range/ accuracy	Optimal range
VOCs^a^	Multipixel metal oxide semiconductor gas sensor	0-60,000 ppb/±10%	<333 ppb
PM_2.5_^b^	Laser-based light scattering particle sensor	0-1000 µg/m^3^/±15 µg/m^3^ or 15%	<15 µg/m^3^

^a^VOC: volatile organic compound.

^b^PM_2.5:_ fine particulate matter.

EMA is a survey approach that uses repeated assessment of participants to capture behaviors and experiences in a real-time context. This methodological approach maintains the ecological validity of reported behaviors and decreases the impact of recall bias because surveys are sent periodically to participants through electronic devices [35]. EMA is particularly useful when trying to understand the context of symptoms or behaviors. Available literature that combines the EMA methodology with asthma research focuses primarily on asthma-related outcomes such as symptoms, control, quality of life, history, and medication adherence and their relationship with stress (ie, perceived stress, mood, hassles, and social support) [36-38]. The ability to time stamp behaviors, events, and experiences and determine whether they present a positive or negative health impact is valuable when seeking to understand the complexity of multiple risk factors and multiple environmental and behavioral interactions. EMA data will be collected electronically using the PiLR EMA software platform, which can be installed on a personal smartphone (iOS or Android) [28]. Data captured will be transmitted to the PiLR EMA platform and stored in a secure database in the cloud. Participants will receive prescheduled notifications asking them to complete small surveys. In addition, we collaborated with PiLR staff to configure the EMA platform to send EMA notifications based on certain logics and triggers (ie, VOC and PM_2.5_ readings exceeding the optimal ranges outlined in Table 1). Researchers can access the PiLR dashboard to monitor participants’ use of the EMA app and review their data. 

The ZEPHYRx Spirometer system allows for laboratory-quality pulmonary function tests (PFTs) at home, such as forced vital capacity (FVC), FEV_1_, FEV_1_/FVC, and flow-volume loop [39]. The system consists of a Food and Drug Administration (FDA)–approved handheld Bluetooth spirometer (MIR Spirobank Smart), and a mobile application that works on a smartphone or tablet device. The spirometer measures 49 × 109 × 21 mm and weighs 60.7 grams. Participants will share PFT results through the ZEPHYRx app, and the research team members will access a Health Insurance Portability and Accountability Act (HIPAA)–compliant portal to view participants’ PFT results in real time. An in-app video coaching feature guides the participant through the PFT maneuver. Each maneuver conducted by the participant creates a time and date–stamped American Thoracic Society standard PFT report accessible through the portal. Deidentified data will be exported from the dashboard as a .CSV file. 

### Participants and Recruitment

#### Participants

This study builds on data collected from the GCAS, a cross-sectional online survey we conducted in 2020 to examine how the COVID-19 pandemic affected the prevalence of disinfectant use among adults with asthma and to assess the association between the frequency of disinfectant use and asthma control as measured by the Asthma Control Test (ACT) [14]. Over 1300 individuals responded. Of those, 93.4% (n=934) indicated they are based in the United States, 78.5% (n=783) are female, about 23% (n=225) indicated a minority race/ethnicity, and 741 agreed to be contacted for follow-up research. About 57% (n=422) used disinfectant/cleaning products ≥5 times per week.

#### Inclusion/Exclusion Criteria

Eligibility criteria include GCAS participants who (1) indicated willingness to be contacted for future research and provided an email address or phone number and (2) reported high use of disinfectant/cleaning products since COVID-19 (≥5 per week) and current ACT ≤19 or ≥2 asthma exacerbations in the past 12 months. The commonly used and validated ACT includes 5 items addressing asthma symptoms, use of rescue medications, and effect of asthma on daily functioning. Responses are summed for total ACT score with scores ≤19 indicating asthma that is not well controlled [40,41]. Non-US residents and non-English speakers are excluded. We will strive to have at least 20% of the sample represent ethnic/racial minority populations.

#### Recruitment

A total of 50 individuals with at least 20%-40% of the sample representing individuals who self-identify as being of a minority race/ethnicity (Hispanic, Black or African American, American Indian or Alaska Native, Asian, Native Hawaiian or other Pacific Islander, Arab, multiracial, or other race) who meet the eligibility criteria will be randomly selected and invited to participate in this study via email or phone. Invites will be sent to selected individuals on a staggered timeline based on response rate and positive participation interest. Participants will be randomized within their participant category (ie, White or minority race/ethnicity). Participants within each category will be randomly assigned an order number using a random number generator in Excel (Microsoft). Participants will be recruited in the order of their random assignment until we have recruited a total of 50.

Using the randomized order assignment, invitations will be sent to 2 to 5 individuals from each group at a time, with the intent of enrolling initially up to 5 participants at any 1 time. If fewer than 5 consent to participate in any wave of invitations, invitations will be sent to the next potential participants in the randomized order assignment. We will scale up to 10 to 15 enrolled participants once we have passed the initial start-up period. The staggered recruitment process will be repeated until 50 individuals including at least 20%-40% minorities have completed study participation. Recruitment will focus on equal numbers from each participant category (ie, 25 each). However, given the smaller initial pool, if we are unable to recruit 25 minority participants, additional participants will be recruited from the White participant pool until a total of 50 participants are enrolled.

Interested individuals will be directed to a Research Electronic Data Capture (REDCap) survey link to confirm willingness to participate, verify eligibility, and provide current contact information. REDCap will be formatted to automatically send 2 additional email invitations 1 week apart if no response is received. If no response is received after the second email invitation and a phone number is provided, a phone call contact will be attempted. If no phone number is provided, a third follow-up email will be sent. A total of 3 contact attempts over a 3-week period with 1 week between each contact will be made to each invited individual. We will randomly select replacements if no response is received after 3 contact attempts, lack of interest is indicated, or inclusion/exclusion criteria are not met.

### Development and Testing of EMA

Four types of EMA surveys will be sent to participants: a daily morning survey (ie, Daily Survey) inquiring whether participants had asthma symptoms and requesting participants use their ZEPHYRx spirometer; 2 daily random surveys (ie, Random Surveys) to assess disinfectant use and asthma symptoms and remind those with asthma symptoms to use their spirometer; and 2 triggered surveys based on elevated VOCs and/or PM_2.5_ levels as captured by Awair Omni. The first triggered survey (ie, Air Quality Event Survey) will be sent to participants as soon as elevated levels of VOCs or PM_2.5_ levels are detected to assess behaviors or environmental changes that may have caused elevation, ask participants to report any asthma symptoms they are experiencing, and remind them to use their spirometer. The second triggered survey (ie, Air Quality Follow-up Survey) will follow 1 hour after the trigger event (ie, elevated VOC or PM_2.5_) and will assess interventions by participants to improve the indoor air quality or reduce their exposure (eg, open a window, leave the room) and any ongoing asthma symptoms. All these surveys were built on the PiLR dashboard by study team members after receiving orientation by PiLR staff. After creating the surveys, research team members were added as “participants” to allow for rigorous testing of the surveys, prompts, skip logics, and so on. Sending EMA surveys triggered by Awair Omni readings required significant programming conducted by PiLR staff. Primary considerations for scheduling the triggered surveys were timing of the survey prompt relative to the event (ie, prompt sent as close to the event as possible) while reducing burden on participant for the number of prompts received (ie, VOC/PM_2.5_ levels may “bounce” above and below the threshold several times within a small interval of time). Real-time data resulted in frequent prompts to respond to the same event, whereas 5-minute averages could result in delay of a few minutes between actual event and the deployment of the survey prompt. Several team members received Awair Omni devices and used them to assess the functionality and potential burden on participants and test the link between Awair Omni readings and EMA triggered surveys to ensure that the proper link is established and real-time data on VOCs and PM_2.5_ are captured that will trigger EMA surveys.

### Development of Participant Guides for Research Tools

How-to guides were developed to describe the set-up and use of each research tool. The goal was to create a seamless guide with step-by-step pictures so a participant could easily follow and pinpoint any missteps. The currently available user guides from the specific companies (ZEPHYRx, Awair Omni, and PiLR EMA) were used as templates, with additional specificity added as needed. For each device, research team members downloaded the app, set up the device, took screenshots of the processes, and wrote step-by-step instructions of the set-up. Issues faced while developing the guides included variations between phone types (eg, iPhone, Android) and software updates that occurred during testing requiring adjustments in the guides. An asthma-focused community advisory board hosted and led by the Chicago Asthma Consortium was utilized to review the guides and provide feedback. This feedback was incorporated into each guide. Suggestions included having study team contact information readily available in case of any issues and using highlighted boxes to relay important information.

### Baseline Measures

Measures that will be administered at baseline via REDCap are outlined in Textbox 1.

**Measures to be administered at baseline via Research Electronic Data Capture (REDCap).**

1. Adult Asthma History includes 5 items adapted from the Behavioral Risk Factor Surveillance System Survey [42] and 3 items from the Airborne Exposures PROMIS (Patient-Reported Outcomes Measurement Information System) Pool V1.0 Dyspnea Airborne Exposure. Four items address participants’ recent contact with health care professionals for asthma. A fifth item addresses changes in routine work/activities due to asthma. An additional 3 items address airborne allergens, pollutants, and living in an area with extreme temperature changes using yes/no response options.
2. Adult Asthma Adherence Questionnaire includes 5 items on following a prescribed asthma medication plan and barriers to following that plan [43].
3. Health Behaviors includes items addressing exercise, use of alcohol, vaping products, marijuana, and cigarettes.
4. Home Environmental Exposure data include time typically spent at home, exposure to residential secondhand smoke, use of hand sanitizer, disinfectants, room where most time is spent in the home, potential asthma triggers in that room (carpeting, vacuuming, flooring), potential asthma triggers in the room participants sleep in, and cleaning products used in the shower/tub, kitchen, and on furniture. Additional data will include frequency of cleaning these areas, exposure to fragrances in the home, use of dryer sheets, presence of plants in the home, use of allergy control covers for pillows and mattress, pets in the home, heating, ventilation, use of a humidifier, having an attached garage, and use of pesticides in the home [44,45].
5. Demographics include where participants live, education level, occupation, ZIP code, health insurance, rent/own home, type of home, number of people in the home, number of bedrooms, 8 items addressing feelings of worry/tiredness/anxiety, self-rated physical health, comorbidities, height/weight, COVID-19 exposure/infection status, COVID-19 vaccine status, face mask, impact of COVID-19 on finances/employment, and an open-ended question to address any additional concerns. 
6. In terms of emotional support the PROMIS_SF_V2 Short Form 4A measures perceived feelings of support, being cared for, and valued (4 items) [46]. Higher scores indicate higher perceived emotional support.
7. Perceived Stress Scale includes 4 items measuring the degree to which situations in one’s life are appraised as stressful. Higher scores indicate an increased level of perceived stress [47].

### Subsequent Measures

#### Seven-Day Check-in

Open-ended questions will be asked to identify any troubleshooting areas, determine if adjustments need to be made, and assess ease of use of the app/devices. Participants will be asked how things went with each of the study tools (Awair Omni indoor air quality monitor, ZEPHYRx spirometer, and EMA). Additional things to be asked include feedback on the set-up materials provided prior to data collection, interactions with research staff, queries if a participant is nonresponsive to EMA prompts, and an open-ended general item about how the study is going. Interactions will be recorded and reviewed by the research team to identify patterns as well as any adjustments that are needed.

#### Participant Feasibility Assessment

At the end of the 14-day data collection period, participants will be asked to respond to a short REDCap survey assessing usability of the Awair Omni indoor air quality monitor (10 items), PiLR EMA Health app (10 items), and the ZEPHYRx home spirometer (10 items), with response ranging from 1 (strongly agree) to 5 (strongly disagree). After each section, there will be an open-ended item for participants to comment on what they liked or did not like about each study tool (Awair Omni air quality monitor, EMA, ZEPHYRx home spirometer). A random sample of 20 participants will be interviewed to further explore these factors using 4 open-ended questions: (1) “How did the study work for you?” (2) “Tell us what you liked/didn’t like about the indoor air quality monitor.” (3) “How did you feel about the integration of the various tools (Awair Omni, ZEPHYRx, and EMA) in the study?” (4) “Is there anything else you’d like to tell us?”

### Data Analysis Plan

Each day of observation will be divided into 4 periods between 6 AM and 10 AM that will be 4 hours each. Data from event-contingent prompts as well as fixed (ie, daily morning and 2 random) prompts will be analyzed. Multiple event–contingent prompts may occur within the same 4-hour window, in which case data will be averaged across all event-contingent prompts during that period. The daily morning survey is scheduled so that it will always occur within the first 4-hour window of the day, and the random prompts are scheduled so only 1 will occur within any of the 4-hour windows.

To address the first aim of this study, we will assess compliance in 4 areas: survey response, spirometry use, interventions to reduce exposure, and air quality monitoring. Survey response compliances will be measured using EMA prompts and defined by the total number of completed prompts of each type out of the number of EMA prompts of each type received. Behavioral compliance to use of the spirometer will be assessed as the number of times the participant used the spirometer out of the number of times the participant was prompted to use it. Compliance to completing interventions to reduce exposure following an air quality event will be assessed using completed follow-up surveys as the number of times the participant reported taking action to reduce exposure out of the number of event-contingent surveys where the participant indicated they were in the home at the time of the event. Air quality monitoring compliance will be assessed as the duration the indoor air quality monitor is used by each participant out of the total study time. Compliance in all 4 areas will be assessed for the entire study period as well as within the 4-hour daily windows.

The quantitative participant assessment of acceptability, appropriateness, and feasibility will be analyzed descriptively. Differences in use and acceptance by participant demographics will be examined to identify factors (eg, age, asthma symptoms, overall health, education) that may make participants more or less likely to use the monitors or respond to prompts. Qualitative interview data will be transcribed and uploaded to Dedoose qualitative analysis software (SocioCultural Research Consultants) for content analysis.

To address the second aim of this study, we will analyze baseline data on the current use and frequency of disinfectants/cleaners and exposure to environmental triggers (eg, secondhand smoke). Real-time self-reported data on disinfectant use throughout the study period obtained through the EMA studies will be analyzed for total use during the study period as well as trends within and across the daily time intervals (eg, levels reporting in morning versus evening survey windows). We will determine the prevalence of disinfectant use and frequency (percent of EMA prompts at which disinfectant use is reported). We will also obtain data from the Awair Omni dashboard to determine the number of times VOC and PM_2.5_ levels exceed optimal levels, average daily levels, average levels per time, and fluctuations in VOC and PM_2.5_. We will identify possible environmental triggers that contribute to high levels of VOCs and PM_2.5_ and event-contingent prompts.

To address the third aim of this study, we will determine the baseline level of asthma control as reported by the ACT. We will use EMA to determine the level of asthma control over the study period as indicated by (A) asthma symptoms reported at each prompt and over the 14 days, (B) number of time periods in which participants report asthma symptoms, and (C) lung function as measured by FEV_1_% predicted within 30 minutes of each event-contingent prompt. Asthma control will also be evaluated using EMA by exploring differences in variability in lung function.

We will use the data collected using the EMA approach to evaluate the fourth aim of this study. EMA data are multilevel, with time windows (level 1) nested in individual participant information (level 2). The exposure variables will include (1) baseline person-level variables such as self-reported use and frequency of disinfectant/cleaner use and PM_2.5_ exposure, (2) time-level exposure frequency variables defined by the number of times VOC and PM_2.5_ levels reach or exceed the threshold for the optimal readings per week, (3) time-level exposure level variables defined by the average scores of VOC and PM_2.5_ levels in the time windows considered in the analysis, and (4) time-level exposure duration variables defined by the total time within the time windows that the VOC and PM_2.5_ levels were elevated. The outcome variables that we will consider include occurrence of any asthma symptoms, inhaler use, and FEV_1_% predicted within 30 minutes from the event-contingent prompt.

We will use multilevel binary logistic regression to examine the associations between the exposure variables and binary outcome variables. We will also use multilevel linear regression to examine associations between the exposure variables and predicted FEV_1_%. We will control for both level 1 variables (time of day, day of week) and level 2 variables (baseline asthma symptoms, demographic characteristics). Using available time stamps from the indoor air quality monitor, EMA, and the spirometer, we will also explore average lag time between an event-contingent prompt and self-reported asthma symptoms using multilevel survival analyses.

## Results

This project received funding in March 2021. The first year of this project has been spent focusing on determining the feasibility and usability of using an EMA to assess self-report of residential environmental exposures, home monitoring or environmental exposures, and lung function. This has involved creating participant research materials and developing and coordinating 4 diverse software platforms: REDCAp surveys, ZEPHYRx spirometer, Awair Omni home air quality monitor, and PiLR EMA. We pilot tested our procedures and began recruitment in March 2022. We anticipate study completion in 2023.

## Discussion

### Summary

This study contributes to our knowledge of the real-time impact of VOCs and PM_2.5_ in the home environment on asthma in several ways. First, it addresses how increased cleaning and disinfecting practices could impact asthma control in people with asthma. This consideration is particularly relevant considering the COVID-19 pandemic, during which people have dramatically increased their use of disinfectants. This study also addresses the methodological shortcomings of previous studies of the home environment of adults with asthma, as we uniquely use real-time collection of symptoms data with EMA to examine the effects of environmental triggers on adult asthma symptoms, real-time objective residential exposure data (VOCs, PM_2.5_), and we collect an objective measure of asthma control with the measurement of lung function.

### Strengths

This study has several strengths. First, it builds on an ongoing study and will be conducted with individuals who have agreed to be recruited for future studies and thus facilitate enrollment. Second, participants will be drawn from a wide geographic area in the United States, which increases broad representation of participants. We will gather real-time environmental exposure data and indicators of asthma control in unregulated indoor home spaces without actual in-person contact. Findings from this study have the potential to impact recommendations for indoor use of disinfectants/cleaners for those with asthma and guide further research.

### Anticipated Challenges and Limitations

There are a number of challenges we anticipate as we launch this feasibility study. First is the presence of the digital divide and the extent to which we may encounter it. The concept of the digital divide acknowledges that communities across nations have differing levels of access to technology, including smartphones, internet, and broadband infrastructure, which may be related to geography (rural versus urban), generational differences, comfort level with technology, and finances. As part of the feasibility design, we will document the impact of this differing access to better address challenges of the digital divide and promote inclusion of varied communities in future studies involving the indoor home environment and technology platforms.

Along with documenting the extent of the digital divide encountered in this study, our team is interested in addressing any ethical concerns that arise with using multiple technology platforms in the indoor home environment. Real-time data collected by Wi-Fi and Bluetooth–enabled devices may bring about unique ethical concerns that must be addressed to ensure privacy and confidentiality. Our team will address any ethical concerns in real time with the guidance of our institutional review board, and we will strive to share best practice when bringing such technology into homes with our research participants as partners in this process. The feasibility design of this study will allow us to purposefully address any challenges that may arise in a larger, powered study.

### Conclusions

Findings from this study will provide preliminary data for a powered study to develop innovative intervention strategies for people with asthma and address the impact of changes in environmental exposures related to COVID-19, thereby enhancing our preparedness for future infectious disease outbreaks. This has implications for risk reduction in people with asthma to help improve asthma self-management using personal monitors and sensors for individually tailored exposure profiles.

## Appendix

Multimedia Appendix 1 External peer-review report from the National Institute of Environmental Health Sciences Special Emphasis Panel - Emerging Research Opportunities in Environmental Health Sciences - Population-based Studies (National Institutes of Health, USA).

## Abbreviations

AC: alternating current
ACT: Asthma Control Test
EMA: ecological momentary assessment
EPA: Environmental Protection Agency
FEV_1_%: forced expiratory volume in 1 second
FVC: forced vital capacity
GCAS: Global COVID-19 Asthma Study
HIPAA: Health Insurance Portability and Accountability Act
NIEHS: National Institute of Environmental Health Sciences
NIH: National Institutes of Health
PFT: pulmonary function test
PM: particulate matter
PM_2.5_: fine particulate matter
PROMIS: patient-reported outcomes measurement information system
REDCap: Research Electronic Data Capture
VOC: volatile organic compound
