# Supplementary material for: Utilizing Real-time Technology to Assess the Impact of Home Environmental Exposures on Asthma Symptoms: Protocol for an Observational Pilot Study
Source: JMIR Res Protoc. 2022 Aug 2;11(8):e39887. doi: 10.2196/39887 (PMC9382544; doi:10.2196/39887)
Supplement: Multimedia Appendix 1 [file resprot_v11i8e39887_app1.pdf]

SUMMARY STATEMENT
(Privileged Communication)

PROGRAM CONTACT:
Bonnie Joubert
984-287-3276
bonnie.joubert@nih.gov

Release Date: 11/11/2020
Revised Date:

Application Number: 1 R21 ES033118-01

Principal Investigators (Listed Alphabetically):

ELDEIRAWI, KAMAL M
POLIVKA, BARBARA J (Contact)

Applicant Organization: UNIVERSITY OF KANSAS MEDICAL CENTER

Review Group: ZES1 LWJ-D (TS)
National Institute of Environmental Health Sciences Special Emphasis Panel
Emerging Research Opportunities in Environmental Health Sciences-Population-based Studies.

Meeting Date: 10/27/2020
Council: JAN 2021
Requested Start: 12/01/2020
RFA/PA: ES19-011
PCC: 2069U99I

Project Title: The Changing COVID-19 Landscape: A Feasibility Study to Capture Momentary Residential Environmental Exposures and Asthma Sypmtoms in Adults
SRG Action: Impact Score:24
Next Steps: Visit https://grants.nih.gov/grants/next_steps.htm
Human Subjects: 30-Human subjects involved - Certified, no SRG concerns
Animal Subjects: 10-No live vertebrate animals involved for competing appl.
Gender: 1A-Both genders, scientifically acceptable
Minority: 1A-Minorities and non-minorities, scientifically acceptable
Age: 3A-No children included, scientifically acceptable

Project Year	Direct Costs Requested	Estimated Total Cost
1	150,000	259,888
2	125,000	216,573
TOTAL	275,000	476,461

ADMINISTRATIVE BUDGET NOTE: The budget shown is the requested budget and has not been adjusted to reflect any recommendations made by reviewers. If an award is planned, the costs will be calculated by Institute grants management staff based on the recommendations outlined below in the COMMITTEE BUDGET RECOMMENDATIONS section.

RESUME AND SUMMARY OF DISCUSSION: This time-sensitive application posits a role of augmented cleaning & disinfecting practices in the home due to SARS-CoV-2 and an unintentional increased incidence/risk of respiratory dysfunction among asthmatics individuals. The premise is that a greater degree of the population is working from home, and resultingly will come into greater contact with VOCs and SHS/$PM_{2.5}$, which are known to exacerbate lung distress. Through the use real-time self-assessment recall during a time of noted distress and the used of FEV monitoring were all considered strengths of this application. The Review Committee also felt the team had the appropriate expertise to carry-out the studies and that they already have in place a small cohort of 50 asthmatics to recruit into the study through an online survey. This along with the use of smartphone technology and that the design will incorporate two-sided testing that could possibly implicate potential beneficial effects through the removal of other lesser known allergens were deemed positive outcomes. Some weaknesses were also raised but were considered minor and did not distract from an otherwise warranted study. Indeed, several panel members expressed some concern with the sensor deployment within the households and that these are located within the more transited areas. It was therefore not clear how translatable the findings would be to other less technological advance households and that there was little discussion in controlling overall data quality. Overall, the Review Committee remained very positive as any reported outcomes would be important and could lead to intervention strategies to reduce asthma attacks among vulnerable populations. A final rating in the high impact range was recommended.

DESCRIPTION (provided by applicant): The global pandemic of coronavirus 2019 (COVID-19) is a substantial cause for concern among individuals with chronic respiratory diseases, including those with asthma. It is estimated that more than 60% of adults with asthma have uncontrolled symptoms and this represents a substantial health and economic impact. Compared to children, adults are nearly five times more likely to die from asthma and the asthma-related death rate is highest among those 65 years and older. Viral infections are a prominent risk factor for asthma exacerbation and, thus, SARS-CoV-2, the virus responsible for COVID-19, is cause for alarm among those diagnosed with asthma. Sheltering-in-place orders and recommendations, physical distancing, wearing face coverings, hand hygiene, and increased cleaning and disinfecting are primary COVID-19 preventative measures advocated. The effects of home-based strategies to prevent COVID-19, specifically increased residential exposure to cleaning/disinfecting agents and particulates on adults with asthma is unknown. Our long-term goal is to characterize the impact of COVID-19 on existing asthma risk factors to develop tailored, home-based asthma interventions that adequately acknowledge COVID-19 and are responsive to the changing home environment and home routines resulting from this pandemic. The aims of this study are: 1) determine the feasibility and usability of: (a) ecological momentary assessment (EMA) to assess self-report residential environmental exposures and asthma symptoms, (b) home monitoring of objective environmental exposures (total volatile organic compounds [VOCs], particulates [$PM_{2.5}$]), and lung function (home spirometry); 2a) assess the frequency and degree of residential environmental exposures (e.g., disinfectants/cleaners, second-hand smoke) via (a) self-reported data, and (b) home monitoring objective measures, 2b) assess the level of asthma control as indicated by self-reported asthma symptoms and lung function; and 3) explore associations of self-reported and objective measures of residential environmental exposures with self-reported and objective measures of asthma control. We will recruit 50 adults with asthma who completed our ongoing online COVID-19 and asthma survey, indicated willingness to be contacted for future studies, reported high use of disinfectant/cleaning products, and have not-well controlled asthma. Participants will receive an indoor air quality monitor and a home spirometer to measure VOCs, $PM_{2.5}$, and FEV1% respectively. EMA will be collected using a personal smartphone and EMA software platform. Participants' will be sent scheduled and random EMA notifications to assess asthma symptoms, environmental exposures, lung function, and mitigation strategies. After the 14-day data collection period, participants will respond to survey items related acceptability, appropriateness, and feasibility. Findings from this feasibility study will support a powered study to address the impact of environmental exposures related to COVID-19

and to enhance preparedness for future infectious disease outbreaks by developing innovative intervention strategies for those with asthma.

PUBLIC HEALTH RELEVANCE: The global pandemic of coronavirus 2019 (COVID-19) has rapidly impacted the US population and is a substantial cause for concern among individuals with chronic respiratory diseases, including those with asthma. Adults appear to be nearly five times more likely to die from asthma and the asthma-related death rate is highest among those 65 years and older. The long term goal of this study is to characterize the impact of COVID-19 on existing asthma risk factors so as to develop tailored, home-based asthma interventions that adequately acknowledge COVID-19 and are responsive to the changing home environment and change home routines resulting from this pandemic.

CRITIQUES: The written critiques of individual reviewers are provided in essentially unedited form below. These critiques were prepared prior to the meeting and may not have been revised afterwards. The "Resume and Summary of Discussion" above summarizes the final opinions of the committee.

CRITIQUE 1

Significance: 2
Investigator(s): 2
Innovation: 2
Approach: 3
Environment: 2

Overall Impact: The applicants propose to remotely collect data on home environmental levels of volatile organic compounds (VOCs) and particulate matter ($PM_{2.5}$) and lung function (spirometry) in adult asthmatics who report low control. The applicants have an extant electronically created cohort from which to recruit participants from across the country. The proposed application raises an interesting question about whether recommended cleaning practices during the COVID-19 pandemic may trigger asthma symptoms. They will use small in-home monitors that will trigger e-contacts with participants to then perform spirometry. The constant access to support by participants for trouble shooting is a strength. The team can do the work as proposed. Their environments will facilitate their success. There is a small concern about the ability to generalize the results to individuals with reduced access to the internet and smart phone technology, but this does not diminish the likely high validity of the results from this study. Overall, the potential impact for this preliminary work on understanding this novel and important question as proposed is quite high.

1. Significance:
Strengths
- This is an important public health and clinical health question. It is highly timely with the pandemic and the increased level of time spent in homes.

Weaknesses
- No major weaknesses are noted.

2. Investigator(s):
Strengths
- The investigative team and Co-Investigators have collaborated previously and have the necessary skills to conduct the work.
- They have the research and clinical expertise.
- This team possesses the skills and experience to trouble-shoot as they move along through the study.

Weaknesses
- No major weaknesses are noted.

3. Innovation:
Strengths
- This is an important question that has likely been lost in all the messaging to improve performance of cleaning practices during this pandemic.
- The data collection methods are novel.

Weaknesses
- The proposed data collection approach may not be suitable for all individuals and this is not discussed. Moreover, validity is the priority and it would be unlikely that any single data collection methodology would work for all asthmatics. This was not discussed in the approach. (very minor weakness).

4. Approach:
Strengths
- They will use an extant cohort of adults with asthma. The cohort will provide some racial and ethnic diversity.
- The protocol is well-detailed.
- The statistical plan is appropriate.
- There is a plan to train the participants on the protocol and plans to provide support to participants in case of equipment failure/issues.

Weaknesses
- It is not clear if the proposed equipment will detect or differentiate all the proposed exposures.
- There is no consideration of assessment of asthma severity (which is a different concept from control).
- Asthmatic individuals have different triggers, and this should be considered in the analyses.

5. Environment:
Strengths
- The environment will support the success of this project.

Weaknesses
- No major weaknesses are noted.

Study Timeline:
Strengths
- Not Applicable

Weaknesses
- Not Applicable

Protections for Human Subjects:
Acceptable Risks and/or Adequate Protections
- No concerns. The use of the term anonymous may not be appropriate in some places in the text, but there is no concern about the plan.

Data and Safety Monitoring Plan (Applicable for Clinical Trials Only):
- Not Applicable (No Clinical Trials)

Inclusion Plans:
- Sex/Gender: Distribution justified scientifically
- Race/Ethnicity: Distribution justified scientifically
- For NIH-Defined Phase III trials, Plans for valid design and analysis: Not Applicable

- Inclusion/Exclusion Based on Age: Distribution justified scientifically
- No concerns with the proposed population are noted.

Vertebrate Animals:
Not Applicable (No Vertebrate Animals)

Biohazards
Not Applicable (No Biohazards)

Resubmission
Not Applicable

Renewal
Not Applicable

Revision
Not Applicable

Applications from Foreign Organizations:
Not Applicable (No Foreign Organizations)

Select Agents:
Not Applicable (No Select Agents)

Resource Sharing Plans:
Not Applicable (No Relevant Resources)

Authentication of Key Biological and/or Chemical Resources:
Not Applicable (No Relevant Resources)

Budget and Period of Support:
Recommended as requested.

CRITIQUE 2

Significance: 3
Investigator(s): 3
Innovation: 3
Approach: 4
Environment: 1

Overall Impact: This proposed exploratory application is developed to test the feasibility and usability of data collection methods and tools including ecological momentary assessment, indoor air pollution monitor, and a low-cost spirometer, to examine the levels of residential environmental exposures and asthma control, and further to assess the impacts of residential environmental exposures on asthma control. The project is moderately innovative as it addresses an emerging issue of increased use of cleaning products and amount time spend at home during this COVID19 pandemic, which might significantly impact asthma control. The major weaknesses of the project are study design and methods. First, although increased use of cleaning products at home will increases exposure to cleaning chemicals, it might also reduce viral infection risk, which further reduces risk of asthma exacerbation. Thus, if the study only emphasizes harmful effects of increased chemical exposure on

asthma but ignores potential benefits of disinfectant use through possibly reducing viral infection on asthma, the conclusion may be biased. Second, while not required some preliminary data and experience in support of participant-controlled data collection process and the procedure for data quality control might be problematic, tempered enthusiasm in the moderate range.

1. Significance:
Strengths
- Asthma causes significant health care burden and economic loss in US.
- The current COVID 19 pandemic has alter cleaning practice and time spending indoor at home, which may impact asthma control. This altered exposure at home during this pandemic receive little attention.

Weaknesses
- Disinfectants/cleaners use at home might significantly reduce viral infections such as COVID19 and influenza, which may cause more worse consequences related to asthma exacerbation. Lack of acknowledge of the potential benefits may be problematic.

2. Investigator(s):
Strengths
- Investigators, Drs. Polivka and Kamal, have track records in environment health and asthma related research. They both are qualified for their roles on the project.
- The research team including biostatistician, nurses and physician processes the complimentary expertise which is required for a successful implementation of this project.

Weaknesses
- Additional expertise in personal air monitoring and indoor air pollution exposure assessment may be helpful.

3. Innovation:
Strengths
- This study is moderately innovative as it addresses a new indoor environmental issue of increased use of cleaning products and amount time spend at home caused by this COVID 19 pandemic. This is the first study to evaluate potential impacts of this altered indoor environment during this pandemic on asthma control.

Weaknesses
- Exposure to disinfectants and indoor particulate matters has been reported to be linked with asthma exacerbation.

4. Approach:
Strengths
- The online survey of Global COVID 19 and Asthma study (GCAS) provides a base for this study.
- Both self-reported data and objective measurements using the devices on residential environmental exposures and asthma control will be obtained.
- Baseline data including asthma history, asthma control test, asthma adherence questionnaire, home environment, emotional support and psychological status are comprehensive and strong.

Weaknesses
- Again, as the investigator noted, viral infections including SARS CoV-2, are a prominent risk factor for asthma exacerbation. To better understand the impacts of home disinfectants/cleaners use on asthma, it requires to know how much viral infections can be potentially prevented by this use. By considering this potential benefit associated with disinfectant use, it may provide better and more comprehensive assessment about the impacts of disinfectants/cleaners use on asthma.

- The procedure of data quality control including instrument calibration and plan for replacing dysfunctional devices, has not been well clarified.
- Lack of preliminary data and experience supports the participant-controlled data collection process.
- Although this is a pilot project, no justification was provided for sample size.

5. Environment:
Strengths
- This is a collaborative project which involves two institutions including the University of Kansas Medical Center and the University of Illinois at Chicago which offers the excellent research environment for this project.

Weaknesses
- No major weaknesses were identified.

Study Timeline:
Strengths
- Not Applicable

Weaknesses
- Not Applicable

Protections for Human Subjects:
Acceptable Risks and/or Adequate Protections

Data and Safety Monitoring Plan (Applicable for Clinical Trials Only):
- Not Applicable (No Clinical Trials)

Inclusion Plans:
- Sex/Gender: Distribution justified scientifically
- Race/Ethnicity: Distribution justified scientifically
- For NIH-Defined Phase III trials, Plans for valid design and analysis: Not Applicable
- Inclusion/Exclusion Based on Age: Distribution justified scientifically

Vertebrate Animals:
Not Applicable (No Vertebrate Animals)

Biohazards:
Not Applicable (No Biohazards)

Resubmission
Not Applicable

Renewal
Not Applicable

Revision
Not Applicable

Applications from Foreign Organizations:
Not Applicable (No Foreign Organizations)

Select Agents:
Not Applicable (No Select Agents)

Resource Sharing Plans:
Acceptable

Authentication of Key Biological and/or Chemical Resources:
Acceptable

Budget and Period of Support:
Recommended as requested.

CRITIQUE 3

Significance: 1
Investigator(s): 1
Innovation: 1
Approach: 2
Environment: 1

Overall Impact: The study seeks to track environmental asthma triggers (i.e., volatile organic compounds [VOCs] and $PM_{2.5}$) in asthma patients' home environment through both self-reported data and objective data from indoor air quality monitors (i.e., Awair Omni). The study aims to recruit 50 individuals who have high use of disinfectant/clean products since COVID-19. Patients' asthma outcome will also be objectively measured using Zephyrx Spirometer for pulmonary function tests (PFTs). The study team is strong with all the necessary expertise. The study design is sensible with careful considerations of different barriers. Minor concern on the deployment of the air quality monitors, as it is unclear whether the entire house will be monitored or only a portion of the house (more likely based on the description, but this also raises potential bias, i.e., participants could be in a different area of the house that does not have increased air quality issues). Overall, this is a competitive and warranted study.

1. Significance:
Strengths
- Increasing use of cleaning and disinfecting products due to COVID-19 could have impact on asthma control among adults. The topic is of high interest.

Weaknesses
- No major weaknesses are noted.

2. Investigator(s):
Strengths
- The investigator team is strong with the necessary expertise.

Weaknesses
- No major weaknesses are noted.

3. Innovation:
Strengths
- Combination of self-reported/EMA and objective measures (from air quality sensors) is novel.

Weaknesses
- No major weaknesses are noted.

4. Approach:
Strengths

- Ongoing preliminary work (i.e., the GCAS) shows feasibility of the proposed study.
- The study design is well-thought out, addressing potential barriers.
- Use validated instruments (e.g., PROMIS).

Weaknesses

- Installation of Awair Omni is up to the participants, which can potentially lead to mismeasurements.
- Further, it looks like only 1 Awair Omni sensor (limited to 1,000 sq. ft) will be installed and the participant will be instructed to have the sensor installed in the room where the participants spend most time. It would be more suitable to cover the entire house (minor, as this is a feasibility study).

5. Environment:
Strengths

- The environment is adequate.

Weaknesses

- No major weaknesses are noted.

Study Timeline:
Strengths

- Not Applicable

Weaknesses

- Not Applicable

Protections for Human Subjects:
Acceptable Risks and/or Adequate Protections

Data and Safety Monitoring Plan (Applicable for Clinical Trials Only):

- Not Applicable (No Clinical Trials)

Inclusion Plans:

- Sex/Gender: Distribution justified scientifically
- Race/Ethnicity: Distribution justified scientifically
- For NIH-Defined Phase III trials, Plans for valid design and analysis: Not Applicable
- Inclusion/Exclusion Based on Age: Distribution justified scientifically

Vertebrate Animals:
Not Applicable (No Vertebrate Animals)

Biohazards:
Not Applicable (No Biohazards)

Resubmission
Not Applicable

Renewal
Not Applicable

Revision
Not Applicable

Applications from Foreign Organizations:
Not Applicable (No Foreign Organizations)

Select Agents:
Not Applicable (No Select Agents)

Resource Sharing Plans:
Not Applicable (No Relevant Resources)

Authentication of Key Biological and/or Chemical Resources:
Not Applicable (No Relevant Resources)

Budget and Period of Support:
Recommended as requested.

THE FOLLOWING SECTIONS WERE PREPARED BY THE SCIENTIFIC REVIEW OFFICER TO SUMMARIZE THE OUTCOME OF DISCUSSIONS OF THE REVIEW COMMITTEE, OR REVIEWERS' WRITTEN CRITIQUES, ON THE FOLLOWING ISSUES:

PROTECTION OF HUMAN SUBJECTS: ACCEPTABLE

INCLUSION OF WOMEN PLAN: ACCEPTABLE

INCLUSION OF MINORITIES PLAN: ACCEPTABLE

INCLUSION ACROSS THE LIFESPAN: ACCEPTABLE

VERTEBRATE ANIMAL (RESUME): NOT APPLICABLE

BIOHAZARDS (RESUME): ACCEPTABLE

APPLICATIONS FROM FOREIGN ORGANIZATIONS (RESUME): NOT APPLICABLE

SELECT AGENTS (RESUME): NOT APPLICABLE

RESOURCE SHARING PLAN (RESUME): ACCEPTABLE

AUTHENTICATION OF KEY BIOLOGICAL AND/OR CHEMICAL RESOURCES (RESUME): NOT APPLICABLE

COMMITTEE BUDGET RECOMMENDATIONS: The budget was recommended as requested.

Footnotes for 1 R21 ES033118-01; PI Name: Polivka, Barbara J

NIH has modified its policy regarding the receipt of resubmissions (amended applications).See Guide Notice NOT-OD-18-197 at https://grants.nih.gov/grants/guide/notice-files/NOT-OD-18-197.html. The impact/priority score is calculated after discussion of an application by averaging the overall scores (1-9) given by all voting reviewers on the committee and multiplying by 10. The criterion scores are submitted prior to the meeting by the individual reviewers assigned to an application, and are not discussed specifically at the review meeting or calculated into the overall impact score. Some applications also receive a percentile

ranking. For details on the review process, see
http://grants.nih.gov/grants/peer_review_process.htm#scoring.

MEETING ROSTER

National Institute of Environmental Health Sciences Special Emphasis Panel
NATIONAL INSTITUTE OF ENVIRONMENTAL HEALTH SCIENCES
Emerging Research Opportunities in Environmental Health Sciences-Population-based Studies.

ZES1 LWJ-D (TS)
10/27/2020

Notice of NIH Policy to All Applicants: Meeting rosters are provided for information purposes only. Applicant investigators and institutional officials must not communicate directly with study section members about an application before or after the review. Failure to observe this policy will create a serious breach of integrity in the peer review process, and may lead to actions outlined in NOT-OD-14-073 at https://grants.nih.gov/grants/guide/notice-files/NOT-OD-14-073.html and NOT-OD-15-106 at https://grants.nih.gov/grants/guide/notice-files/NOT-OD-15-106.html, including removal of the application from immediate review.

CHAIRPERSON(S)

FACTOR-LITVAK, PAM R., PHD
ASSOCIATE DEAN FOR RESEARCH
DEPARTMENT OF EPIDEMIOLOGY
MAILMAN SCHOOL OF PUBLIC HEALTH
COLUMBIA UNIVERSITY
NEW YORK, NY 10032

MEMBERS

BARON, SHERRY L., MD
PROFESSOR
BARRY COMMONER CENTER FOR HEALTH
AND THE ENVIRONMENT
QUEENS COLLEGE
CITY UNIVERSITY OF NEW YORK
QUEENS, NY 11367

BIAN, JIANG, PHD
ASSOCIATE PROFESSOR
DEPARTMENT OF HEALTH OUTCOMES
AND BIOMEDICAL INFORMATICS
COLLEGE OF MEDICINE
UNIVERSITY OF FLORIDA
GAINESVILLE, FL 32610

HORNEY, JENNIFER A., PHD
FOUNDING DIRECTOR AND PROFESSOR
EPIDEMIOLOGY PROGRAM AND CORE FACULTY
DISASTER RESEARCH CENTER
COLLEGE OF HEALTH SCIENCES
UNIVERSITY OF DELAWARE
NEWARK, DE 19716

JASPERS, ILONA, PHD
PROFESSOR
DEPARTMENT OF PEDIATRICS
CENTER FOR ENVIRONMENTAL MEDICINE
UNIVERSITY OF NORTH CAROLINA AT CHAPEL HILL
CHAPEL HILL, NC 27599

LOWE, SARAH RYAN, PHD
ASSISTANT PROFESSOR
DEPARTMENT OF PSYCHOLOGY
COLLEGE OF HUMANITIES AND SOCIAL SCIENCES
MONTCLAIR STATE UNIVERSITY
MONTCLAIR, NJ 07043

SRIVASTAVA, SANJAY, PHD
PROFESSOR AND DISTINGUISHED UNIVERSITY SCHOLAR
DEPARTMENT OF MEDICINE
DIVISION OF ENVIRONMENTAL MEDICINE
SCHOOL OF MEDICINE
UNIVERSITY OF LOUISVILLE
LOUISVILLE, KY 40202

WEGIENKA, GANESA REBECCA, PHD
EPIDEMIOLOGIST/ASSOCIATE SCIENTIST
CENTER FOR ALLERGY, ASTHMA AND IMMUNOLOGY
RESEARCH
DEPARTMENT OF PUBLIC HEALTH SCIENCES
HENRY FORD HEALTH SYSTEM
DETROIT, MI 48202

XU, XIAOHUI, PHD
ASSOCIATE PROFESSOR
DEPARTMENT OF EPIDEMIOLOGY AND BIOSTATISTICS
SCHOOL OF PUBLIC HEALTH
TEXAS A&M UNIVERSITY
COLLEGE STATION, TX 77843

SCIENTIFIC REVIEW OFFICER

WORTH, LEROY, PHD
SCIENTIFIC REVIEW OFFICER
SCIENTIFIC REVIEW BRANCH
DIVISION OF EXTRAMURAL RESEARCH AND TRAINING
NATIONAL INSTITUTE OF ENVIRONMENTAL
HEALTH SCIENCES
RESEARCH TRIANGLE PARK, NC 27709

EXTRAMURAL SUPPORT ASSISTANT

JONES, DEBORAH A.
EXTRAMURAL SUPPORT ASSISTANT
SCIENTIFIC REVIEW BRANCH
DIVISION OF EXTRAMURAL RESEARCH AND TRAINING
NATIONAL INSTITUTE OF ENVIRONMENTAL
HEALTH SCIENCES
RESEARCH TRIANGLE PARK, NC 27709

PROGRAM REPRESENTATIVE

CHAMBERLAIN, TOCCARA, MA
PROGRAM ANALYST
POPULATION HEALTH BRANCH
DIVISION OF EXTRAMURAL RESEARCH AND TRAINING
NATIONAL INSTITUTE OF ENVIRONMENTAL
HEALTH SCIENCES
RESEARCH TRIANGLE PARK, NC 27709

GRANTS MANAGEMENT REPRESENTATIVE

GREER, JENNY L., MA
BRANCH CHIEF
GRANTS MANAGEMENT BRANCH
DIVISION OF EXTRAMURAL RESEARCH AND TRAINING
NATIONAL INSTITUTE OF ENVIRONMENTAL
HEALTH SCIENCES
RESEARCH TRIANGLE PARK, NC 27709

Consultants are required to absent themselves from the room during the review of any application if their presence would constitute or appear to constitute a conflict of interest.
